# Influence of the naturally occurring human *CASP1* variant L265S on subcellular distribution and pyroptosis

**DOI:** 10.1186/1546-0096-13-S1-O61

**Published:** 2015-09-28

**Authors:** S Rabe, MC Heymann, R Stein, F Kapplusch, S Russ, F Schulze, S Winkler, W Staroske, A Rösen-Wolff, SR Hofmann

**Affiliations:** 1 Technische Universität Dresden, Department of Pediatrics, Medizinische Fakultät Carl Gustav Carus, Dresden, Germany; 2 Technische Universität Dresden, Biotechnology Center, Dresden, Germany

## Introduction

Patients with unexplained recurrent febrile episodes and *CASP1* variants suffer from systemic sterile inflammation despite altered enzymatic activity of procaspase-1 and reduced IL-1β release. Most recent findings from our group indicate that the proinflammatory effects of *CASP1* variants with reduced or abrogated enzymatic activity could be due to receptor interacting protein kinase 2 (RIP2) mediated increase of NF-kB activation. These findings are additionally supported by a trend to elevated IL-6 and TNF-α expression in patients with *CASP1* variants.

## Objectives

The objective of this project is the identification of possible subcellular mechanisms how the *CASP1-*L265S variant influences proinflammatory cell death (pyroptosis) and IL-1β secretion.

## Methods

We established an in vitro model of a virally transduced human monocytic cell line (THP-1 with shRNA knock-down of endogenous procaspase-1), expressing either wild type or enzymatically inactive (L265S) procaspase-1 fusion-reporter proteins and characterized them after NLRP3-inflammasome stimulation. Using confocal microscopy and in vivo live cell imaging we analyzed the subcellular distribution of fluorescently labeled procaspase-1 wildtype and variant as well as the interaction with ASC.

## Results

First results revealed a disturbed nuclear localization of the *CASP1*-L265S variant compared to procaspase-1 wildtype. Furthermore, *CASP1*-L265S variant revealed a strongly decreased pyroptosome formation and a less intense interaction with ASC (apoptosis-associated speck-like protein containing a CARD) after NLRP3-stimulation. Variant procaspase-1 L265S and ASC formed smaller pyroptosomes than wildtype procaspase-1and ASC.

## Conclusion

Those findings suggest a model, in which variant procaspase-1 L265S impairs nuclear localization, pyroptosome formation and ASC-interaction, leading all together to reduced IL-1β production and secretion.

